# Sarcopenic obesity is associated with impaired physical function and mortality in older patients with heart failure: insight from FRAGILE-HF

**DOI:** 10.1186/s12877-022-03168-3

**Published:** 2022-07-05

**Authors:** Hiroshi Saito, Yuya Matsue, Kentaro Kamiya, Nobuyuki Kagiyama, Daichi Maeda, Yoshiko Endo, Hidenao Ueno, Kenji Yoshioka, Akira Mizukami, Kazuya Saito, Yuki Ogasahara, Emi Maekawa, Masaaki Konishi, Takeshi Kitai, Kentaro Iwata, Kentaro Jujo, Hiroshi Wada, Masaru Hiki, Taishi Dotare, Tsutomu Sunayama, Takatoshi Kasai, Hirofumi Nagamatsu, Tetsuya Ozawa, Katsuya Izawa, Shuhei Yamamoto, Naoki Aizawa, Kazuki Wakaume, Kazuhiro Oka, Shin-ichi Momomura, Tohru Minamino

**Affiliations:** 1 Department of Cardiovascular Biology and Medicine, Juntendo University Graduate School of Medicine, 2-1-1 Hongo, Bunkyo-ku, Tokyo, 113-8421 Japan; 2 Department of Rehabilitation, Kameda Medical Center, Chiba, Japan; 3 Cardiovascular Respiratory Sleep Medicine, Juntendo University Graduate School of Medicine, Tokyo, Japan; 4 Department of Rehabilitation, School of Allied Health Sciences, Kitasato University, Kanagawa, Japan; 5 Department of Cardiology, The Sakakibara Heart Institute of Okayama, Okayama, Japan; 6 Department of Digital Health and Telemedicine R&D, Juntendo University, Tokyo, Japan; 7 Department of Cardiovascular Biology and Medicine, Juntendo University Faculty of Medicine, Tokyo, Japan; 8 Department of Cardiology, Osaka Medical and Pharmaceutical University, Osaka, Japan; 9 Department of Cardiology, Kameda Medical Center, Chiba, Japan; 10 Department of Rehabilitation, The Sakakibara Heart Institute of Okayama, Okayama, Japan; 11 Department of Nursing, The Sakakibara Heart Institute of Okayama, Okayama, Japan; 12 Department of Cardiovascular Medicine, Kitasato University School of Medicine, Kanagawa, Japan; 13 Division of Cardiology, Yokohama City University Medical Center, Kanagawa, Japan; 14 Department of Cardiovascular Medicine, Kobe City Medical Center General Hospital, Hyogo, Japan; 15 Department of Rehabilitation, Kobe City Medical Center General Hospital, Hyogo, Japan; 16 Department of Cardiology, Nishiarai Heart Center Hospital, Tokyo, Japan; 17 Department of Cardiovascular Medicine, Saitama Medical Center, Jichi Medical University, Saitama, Japan; 18 Department of Cardiology, Tokai University School of Medicine, Tokyo, Japan; 19 Department of Rehabilitation, Odawara Municipal Hospital, Kanagawa, Japan; 20 Department of Rehabilitation, Kasukabe Chuo General Hospital, Saitama, Japan; 21 Department of Rehabilitation, Shinshu University Hospital, Nagano, Japan; 22 Department of Cardiovascular Medicine, Nephrology and Neurology, University of Ryukyus, Okinawa, Japan; 23 Department of Rehabilitation, Kitasato University Medical Center, Saitama, Japan; 24 Department of Rehabilitation, Saitama Citizens Medical Center, Saitama, Japan; 25 Saitama Citizens Medical Center, Saitama, Japan; 26 Japan Agency for Medical Research and Development-Core Research for Evolutionary Medical Science and Technology (AMED-CREST), Japan Agency for Medical Research and Development, Tokyo, Japan

**Keywords:** Sarcopenic obesity, Physical function, Mortality, Older adults, Heart failure

## Abstract

**Background:**

The purpose of this study was to clarify the prevalence, association with frailty and exercise capacity, and prognostic implication of sarcopenic obesity in patients with heart failure.

**Methods:**

The present study included 779 older adults hospitalized with heart failure (median age: 81 years; 57.4% men). Sarcopenia was diagnosed based on the guidelines by the Asian Working Group for Sarcopenia. Obesity was defined as the percentage of body fat mass (FM) obtained by bioelectrical impedance analysis. The FM cut-off points for obesity were 38% for women and 27% for men. The primary endpoint was 1-year all-cause death. We assessed the associations of sarcopenic obesity occurrence with the short physical performance battery (SPPB) score and 6-minute walk distance (6MWD).

**Results:**

The rates of sarcopenia and obesity were 19.3 and 26.2%, respectively. The patients were classified into the following groups: non-sarcopenia/non-obesity (58.5%), non-sarcopenia/obesity (22.2%), sarcopenia/non-obesity (15.3%), and sarcopenia/obesity (4.0%). The sarcopenia/obesity group had a lower SPPB score and shorter 6MWD, which was independent of age and sex (coefficient, − 0.120; *t*-value, − 3.74; *P* < 0.001 and coefficient, − 77.42; *t*-value, − 3.61; *P* < 0.001; respectively). Ninety-six patients died during the 1-year follow-up period. In a Cox proportional hazard analysis, sarcopenia and obesity together were an independent prognostic factor even after adjusting for a coexisting prognostic factor (non-sarcopenia/non-obesity vs. sarcopenia/obesity: hazard ratio, 2.48; 95% confidence interval, 1.22–5.04; *P* = 0.012).

**Conclusion:**

Sarcopenic obesity is a risk factor for all-cause death and low physical function in older adults with heart failure.

**Trial registration:**

University Hospital Information Network (UMIN-CTR: UMIN000023929).

**Supplementary Information:**

The online version contains supplementary material available at 10.1186/s12877-022-03168-3.

## Background

Sarcopenia is defined by decreased skeletal muscle mass and muscle strength or walking speed in older adults. The prevalence of sarcopenia varies widely [1–3] because of different definitions or measurement methods used or different populations studied [4, 5]. Sarcopenia is strongly related to aging, although it can also be caused by other diseases [4]; heart failure (HF) is a major cause of secondary sarcopenia, and numerous studies have described an association of sarcopenia with poor prognosis in patients with HF [6–8]. Sarcopenic obesity is a condition in which sarcopenia and obesity coexist; in other words, the lean body mass is reduced in a context of excess adiposity [5]. A recent meta-analysis reported that sarcopenic obesity is associated with significantly higher all-cause mortality for older people [6]; moreover, the prevalence was found to be 18.5% in 168 men with HF [7]. However, no comprehensive large-scale study has hitherto been conducted on sarcopenic obesity in older patients with HF, and therefore its clinical implications remain unclear. Evaluating sarcopenic obesity in patients with HF is essential, as numerous observational studies have reported that obesity may be associated with better prognosis in patients diagnosed with HF [8, 9]. The purpose of this study was to clarify the prevalence, association with frailty and exercise capacity, and prognostic implication of sarcopenic obesity in patients with HF. We investigated the association between sarcopenic obesity status and the short physical performance battery (SPPB) score/6-minute walk distance (6MWD). Furthermore, we examined the prognostic implication of sarcopenic obesity using all-cause death as primary outcome and cardiovascular death (CVD) and non-CVD within 1 year as secondary outcomes.

## Methods

### Study design and patient population

The present study was a post-hoc sub-analysis of FRAGILE-HF (the prevalence and prognostic value of physical and social frailty in geriatric patients hospitalized for HF: a multicentre prospective cohort study) [10], which was a prospective multicentre registry focusing on the prevalence and prognostic impact of multi-domain frailty (physical frailty, social frailty, and cognitive dysfunction) for patients hospitalized with HF. The detailed study design has been published elsewhere [10]. Briefly, all consecutive patients who were hospitalized due to HF decompensation, aged ≥65 years, and could ambulate were evaluated for eligibility from September 2016 to March 2018. The decompensation of HF was diagnosed based on the Framingham criteria [11].

Patients with the following criteria were excluded: (i) previous heart transplantation or current left ventricular assist device use, (ii) undergoing chronic peritoneal dialysis or haemodialysis, (iii) current acute myocarditis, (iv) missing brain natriuretic peptide (BNP) or N-terminal pro BNP (NT-proBNP) data, and (v) BNP level < 100 pg/mL or NT-proBNP level < 300 pg/mL at admission. Among 15 participating hospitals in Japan, 8 were university hospitals, and 7 were non-university teaching hospitals. Physical findings, echocardiography, and blood samples were taken in stable-state before discharge.

Our study complies with the principles of the Declaration of Helsinki and the Japanese Ethical Guideline for Medical and Health Research involving Human Subjects. The study protocol was approved by the ethics committee of each participating hospital (the Sakakibara Heart Institute of Okayama, Research ethics committee [no approval number, approval date: August 18th, 2016], Juntendo University Graduate School of Medicine, Research ethics committee [approval number: 16–150]; Nishiarai Heart Center Hospital, Research ethics committee [approval number: 2016–03]; Kitasato University, Research ethics committee [approval number: B16–107]; Kameda Medical Center, Research ethics committee [approval number: 16–080]; Yokohama City University Medical Center, Research ethics committee [approval number: B161000019]; Kobe City Medical Center General Hospital, Research ethics committee [approval number: zn170114]; Saitama Medical Center, Research ethics committee [approval number: S16–035]; Tokai University School of Medicine, Research ethics committee [approval number: 16R122]; Odawara Municipal Hospital, Research ethics committee [approval number: 2016–01]; Kasukabe Chuo General Hospital, Research ethics committee [approval number: 1702–4]; Shinshu University Hospital, Research ethics committee [approval number: 3565]; University of the Ryukyus, Research ethics committee [approval number: 365]; Kitasato University Medical Center, Research ethics committee [approval number: 28–37]; Saitama Citizens Medical Center, Research ethics committee [no approval number, approval date: December 26th, 2016]). The study information is available in the University Hospital Information Network (UMIN-CTR, unique identifier: UMIN000023929).

### Definition of sarcopenia and obesity

The assessment of sarcopenia and obesity was performed by trained personnel before discharge when patients were in a state of compensation and euvolemia. Sarcopenia was defined according to the Asian Working Group for Sarcopenia (AWGS) criteria [12]; this definition is the most widely used and accepted definition in the Asian population [13]. Following the AWGS criteria, participants were diagnosed with sarcopenia when low muscle strength or physical performance coexisted with low skeletal muscle mass. We defined low muscle strength as handgrip strength < 26 kg for men and < 18 kg for women; low physical performance was defined as a 4-m walk time ≤ 0.8 m/s for both sexes [12]. We measured the usual gait speed by evaluating the 4-m walk time. Handgrip strength was measured using a digital dynamometer (TKK 5101 Grip-D; Takei, Tokyo, Japan). Patients were instructed to sit on a chair with the elbow joint flexed at 90 ^°^ during dynamometry. The patients squeezed the dynamometer gradually and continuously for 3 s, while, performing the test with the right and left hands in turn. The highest handgrip strength values on the right and left sides were averaged and expressed as an absolute value (kg) [14]. We used bioelectrical impedance analysis (BIA; BC-622; Tanita, Tokyo, Japan) to measure appendicular skeletal muscle mass. Appendicular skeletal muscle mass index was calculated as the sum of muscle masses in the extremities divided by the height squared (kg/m^2^). The cut-off values were ≤ 7.0 kg/m^2^ for men and ≤ 5.7 kg/m^2^ for women [13]. The AWGS encourages researchers to provide the coefficient of variance as well as inter- and intra-examiner reliabilities, whenever possible, to facilitate subsequent international comparisons.

Obesity was defined based on the percentage of body fat mass (FM) obtained from the BIA. Cut-off points for obesity were 38 and 27% FM for women and men, respectively [15].

### Measurement of physical function and frailty

The SPPB score and 6MWD were evaluated by experienced physical therapists and/or HF specialists. The SPPB score consists of three physical performance tests to assess each frailty domain of balance (static standing balance), gait speed test (4-m walk time), and weakness (time to complete five repeated chair stands) [16]. Each test is scored from 0 to 4, for a total score of 0 to 12. For the balance test, participants were instructed to maintain their feet in side-by-side, semi-tandem, and tandem positions for 10 s each. For the gait speed assessment test, participant usual speed was measured during a 4-m walk. For the chair stand test, participants were instructed to stand up and sit down five times as quickly as possible. The 6MWD was assessed in an unobstructed hallway based on the guideline [17]. Patients were instructed to walk as fast as possible between two points positioned 30 m apart, and the distance walked in 6 min was recorded. During the 6-minute walk test, patients were allowed to slow down, stop, and rest as needed, but were instructed to resume walking as soon as possible; moreover, they were permitted the use of an assistive device, if needed.

Physical frailty was evaluated using the Fried phenotype criteria, which are considered to be the validated standard [18]. The criteria were originally derived from community-dwelling older people in the Cardiovascular Health Study, and externally validated in the Woman’s Health and Aging study [19]. In the criteria, physical frailty was assessed based on the following five components: slowness (gait speed), weakness (handgrip), weight loss, exhaustion, and low physical activity. Low activity was defined by asking the following questions: (i) how many days a week do you do low−/moderate-intensity exercise? (ii) how many days a week do you do regular exercise/sports? If “No” was the answer to both questions, low physical activity was diagnosed [20].

### Outcomes

We prospectively evaluated the prognosis of patients within 1 year of discharge. Data were collected up to March 2019. The endpoint of this study was all-cause death within 1 year of discharge. The incidences of CVD and non-CVD within 1 year of discharge were also obtained. The incidences of CVD and non-CVD were assessed using the American College of Cardiology/American Heart Association key data elements [21]. After discharge, the patients were followed up in outpatient clinics or other medical facilities at least every 3 months. For those who did not undergo follow-up in clinics, prognostic data were obtained using telephone interviews and an analysis of medical records in the different facilities.

### Statistical analysis

Data were expressed as mean ± standard deviation for normally distributed variables, and as median with interquartile range (IQR) for non-normally distributed data. Categorical data are expressed as frequencies and percentages. When necessary, variables were log-transformed for further analyses. Group differences were evaluated using Student’s *t*-test or the Mann-Whitney *U*-test for continuous variables and the chi-squared or Fisher’s exact tests for categorical variables. Univariate and multivariate linear regression analyses were performed to determine the association between sarcopenic obesity, SPPB score, and 6MWD using age and sex as adjustment variables in multivariate models. In linear regression analysis, the regression coefficient, *t*-value and *P* value are presented. The *t*-value is defined as the regression coefficient divided by its standard error, and it indicates how many times of standard error the coefficient is away from zero. The larger the *t*-value, the more certain the coefficient is not zero. We used the *t*-value for the computation of the *P*-value. Moreover, analysis of covariance (ANCOVA) was used to compare between the groups in terms of SPPB score and 6MWD adjusted by age and sex. Kaplan-Meier survival curves were constructed and compared the event rates using log-rank tests to evaluate the association between sarcopenic obesity status and prognosis. We also compared the prognoses between the excluded and included patients in the current study. For the outcome of all-cause death, we used the Meta-analysis Global Group in Chronic Heart Failure (MAGGIC) risk score [22] and log-transformed BNP values as adjustment variables in the multivariate Cox model. The MAGGIC risk score consists of the patients’ age, sex, left ventricular ejection fraction, body mass index (BMI), creatinine, New York Heart Association class, smoking status, complications (diabetes and chronic obstructive pulmonary disease), HF history, and medication use (angiotensin-converting enzyme inhibitor, angiotensin receptor blocker, and beta-blocker). This risk score has been validated to discriminate the results for mortality and calibration in the Japanese population, even after adding BNP levels [23]. A two-tailed *P* value < 0.05 was considered statistically significant. Statistical analyses were performed using R version 3.5.2 (R Foundation for Statistical Computing, Vienna, Austria; ISBN 3–900,051–07-0, URL http://www.R-project.org).

## Results

### Study patients

During the study period, 1332 hospitalized patients were registered in the FRAGILE-HF study. After excluding 553 patients due to missing data regarding sarcopenia or obesity status, the remaining 779 patients were included in the analysis. There was no difference in the Kaplan-Meier analysis findings between the excluded and included patients (Supplemental Fig. 1). The median age of the study population was 81 years (IQR: 74–86 years), and 57.4% of participants were men. The study population was classified into four groups as follows: non-sarcopenia/non-obesity (*n* = 456, 58.5%); non-sarcopenia/obesity (*n* = 173, 22.2%); sarcopenia/non-obesity (*n* = 119, 15.3%); and sarcopenia/obesity (*n* = 31, 4.0%). Participant baseline characteristics are shown in Table 1. The sarcopenia/obesity group was associated with older age, a higher proportion of men, and a higher proportion of frail patients, as well as higher levels of haemoglobin, haematocrit, serum albumin, total cholesterol, and triglycerides.

**Table 1:** Baseline characteristics based on sarcopenia/obesity status

Variables	Non-sarcopenia/Non-obesity *n* = 456	Non-sarcopenia/Obesity *n* = 173	Sarcopenia/Non-obesity *n* = 119	Sarcopenia/Obesity *n* = 31	*P* value
Age (years)	81 [74–86]	79 [72–85]	82 [76–86]	84 [80–88]	0.003
Male sex (%)	238 (52)	101 (58)	81 (68)	27 (87)	< 0.001
BMI (kg/m^2^)	20.7 ± 2.7	25.7 ± 3.6	18.1 ± 2.4	21.6 ± 3.1	< 0.001
Body fat mass (%)	23.6 ± 7.5	36.7 ± 7.6	21.4 ± 7.2	35.1 ± 8.1	< 0.001
ASMI (kg/m^2^)	7.24 [6.47–8.20]	7.66 [7.02–8.61]	6.04 [5.48–6.55]	6.02 [5.37–6.62]	< 0.001
NYHA class III/IV (%)	54 (12)	20 (12)	13 (11)	3 (10)	0.998
Systolic blood pressure (mmHg)	116 ± 167	115 ± 16	112 ± 16	112 ± 14	0.086
Diastolic blood pressure (mmHg)	63 ± 11	64 ± 10	60 ± 11	63 ± 11	0.027
Heart rate (bpm)	70 ± 14	71 ± 15	72 ± 13	69 ± 18	0.680
Left ventricular ejection fraction (%)	46 ± 16	46 ± 17	45 ± 18	43 ± 18	0.585
Comorbidities (%)					
Atrial fibrillation	202 (44)	88 (51)	45 (38)	15 (48)	0.163
Coronary artery disease	148 (33)	70 (41)	40 (34)	8 (26)	0.202
COPD	43 (9)	24 (14)	16 (13)	7 (23)	0.071
Diabetes	138 (30)	81 (47)	39 (33)	9 (29)	0.001
Hypertension	323 (71)	136 (79)	78 (66)	24 (77)	0.073
Physical frailty by Fried criteria	219 (51)	84 (52)	82 (71)	22 (76)	< 0.001
Laboratory data at discharge					
Haemoglobin (g/dL)	11.8 ± 2.0	12.3 ± 2.1	12.0 ± 2.0	12.6 ± 2.5	0.004
Haematocrit (%)	36.2 ± 5.9	37.8 ± 6.0	36.6 ± 5.8	38.2 ± 7.1	0.012
Albumin (g/dL)	3.4 ± 0.5	3.5 ± 0.5	3.4 ± 0.4	3.5 ± 0.4	0.043
ALT (U/L)	16 [11–24]	17 [11–25]	17 [11–28]	14 [11–20]	0.516
AST (U/L)	22 [17–29]	22 [17–28]	23 [17–31]	21 [16–26]	0.529
Creatinine (mg/dL)	1.30 ± 0.71	1.45 ± 0.72	1.29 ± 0.66	1.36 ± 0.64	0.080
BUN (mg/dL)	25 [19–33]	26 [19–36]	26 [20–37]	28 [19–35]	0.326
eGFR (mL/min/1.73m^2^)	56 ± 22	50 ± 20	56 ± 21	54 ± 19	0.016
BNP (pg/mL)	262 [129–460]	234 [102–509]	284 [168–560]	325 [153–461]	0.171
Total cholesterol (mg/dL)	165 ± 39	153 ± 34	159 ± 33	170 ± 40	0.003
HDL cholesterol (mg/dL)	50 ± 15	45 ± 13	49 ± 15	49 ± 14	0.008
Triglyceride (mg/dL)	92 ± 38	102 ± 44	88 ± 41	109 ± 64	0.004
LDL cholesterol (mg/dL)	96 ± 32	87 ± 30	92 ± 31	100 ± 35	0.024
Sodium (mEq/L)	140 ± 3	140 ± 4	137 ± 4	139 ± 3	< 0.001
Potassium (mEq/L)	4.4 ± 0.5	4.4 ± 0.5	4.4 ± 0.5	4.4 ± 0.5	0.693
Prescription at discharge (%)					
ACE-I/ARB	307 (67)	133 (77)	81 (68)	20 (65)	0.115
Beta-blocker	324 (71)	135 (78)	94 (79)	23 (74)	0.168
Mineralocorticoid receptor antagonist	44 (10)	14 (8)	7 (6)	1 (3)	0.395
Loop diuretics	258 (57)	105 (61)	69 (58)	19 (61)	0.795

Values are expressed as median [interquartile range], n (%), or mean ± standard deviation

*ACE-I* angiotensin-converting enzyme inhibitor, *ALT* alanine aminotransferase, *ARB* angiotensin receptor blocker, *ASMI* appendicular skeletal muscle mass, *AST* aspartate aminotransferase, *BMI* body mass index, *BNP* brain natriuretic peptide, *bpm* beats per minute, *BUN* blood urea nitrogen, *COPD* chronic obstructive pulmonary disease, *eGFR* estimated glomerular filtration rate, *HDL* high-density lipoprotein, *LDL* low-density lipoprotein, *NYHA* New York Heart Association

### Physical function and functional capacity

**Fig. 1:**
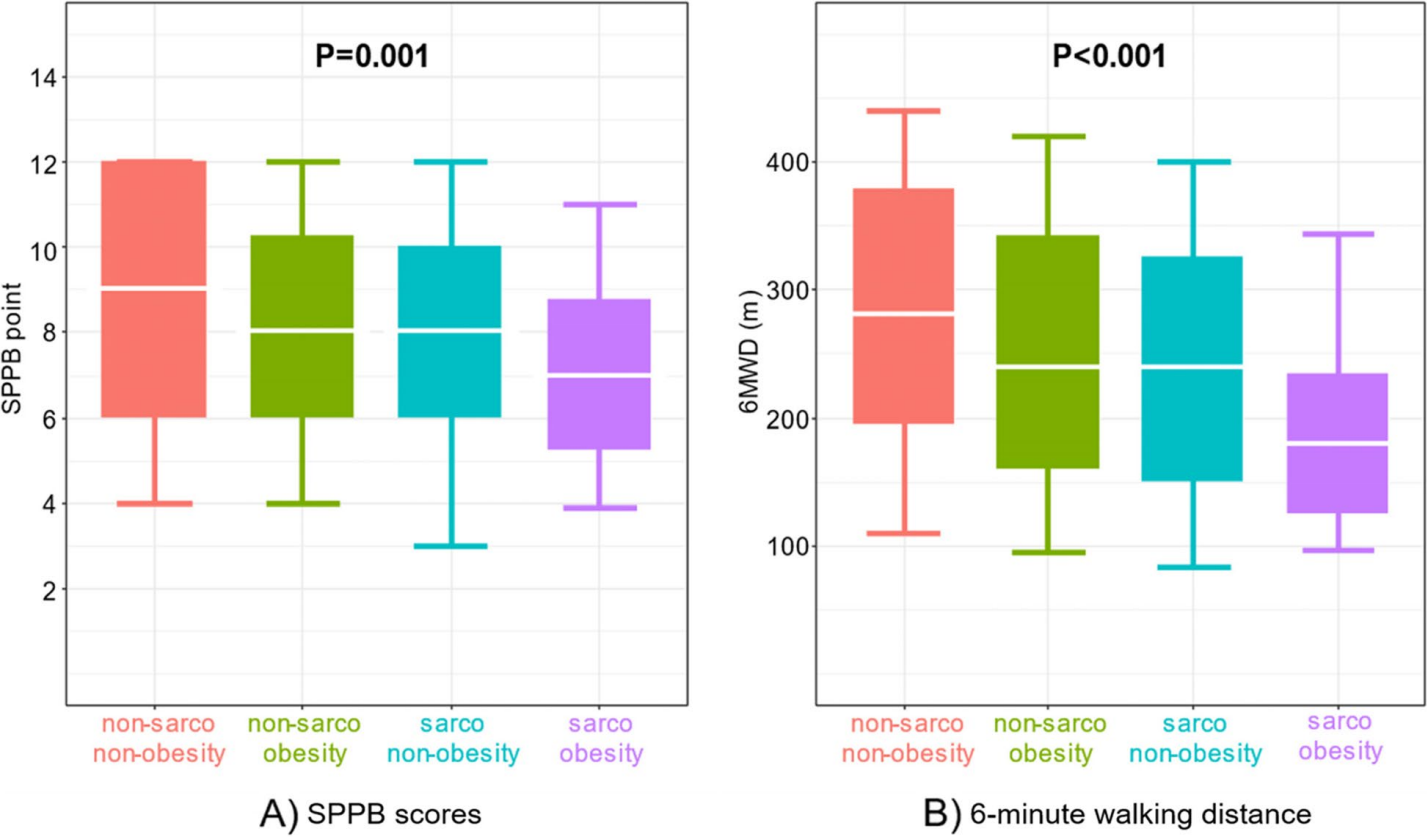
Short physical performance battery scores (**A**) and 6-minute walking distance (**B**) based on the presence or absence of sarcopenia and obesity. The top and bottom of each box show the 75th and 25th percentiles, respectively, and the horizontal lines within each box indicate the median. The whiskers represent the 10th and 90th percentiles. 6MWD, 6-minute walking distance; SPPB, short physical performance battery

SPPB scores were significantly lower in the sarcopenia/obesity group than in other groups (Fig. 1A). The median SPPB scores were 9 [IQR, 6–12], 8 [IQR, 6–10], 8 [IQR, 6–10], and 7 [IQR, 5–9] in patients with non-sarcopenia/non-obesity, non-sarcopenia/obesity, sarcopenia/non-obesity, and sarcopenia/obesity (*P* = 0.001), respectively. Compared to the reference group (non-sarcopenia/non-obesity group), patients with sarcopenia/obesity had significantly lower SPPB scores in the linear regression analysis after adjusting for age and sex (coefficient, − 0.120; *t*-value, − 3.74; *P* < 0.001) (Table 2). Similarly, patients with sarcopenia/obesity had significantly shorter 6MWD than those in other groups (Fig. 1B). The median values of 6MWD were 280 [IQR, 195–379] m, 240 [IQR, 160–342] m, 240 [IQR, 150–326] m, and 180 [IQR, 125–235] m in patients with non-sarcopenia/non-obesity, non-sarcopenia/obesity, sarcopenia/non-obesity, and sarcopenia/obesity (*P* < 0.001), respectively. Linear regression analysis revealed that patients with sarcopenia/obesity had significantly shorter 6MWD than those with non-sarcopenia/non-obesity after adjusting for age and sex (coefficient, − 77.42; *t*-value, − 3.61; *P* < 0.001) (Table 2). In addition, patients with sarcopenia/non-obesity had significantly higher SPPB scores and longer 6MWD than those with sarcopenia/obesity after adjusting for age and sex (SPPB score: coefficient 4.23, *t*-value 2.14, *P* = 0.032; 6MWD: coefficient 45.64, *t*-value 2.00, *P* = 0.045). Moreover, ANCOVA showed that sarcopenia/obesity occurrence was significantly associated with lower SPPB scores and 6MWD than those with non-sarcopenia/non-obesity after adjustment for age and sex (Supplemental Table 1).

**Table 2:** Univariate and multivariate linear regression analysis between sarcopenia/obesity status and physical functions

**SPPB**	**Unadjusted model**			Adjusted model*		
	**Coefficient**	***t*** **value**	***P*** **value**	**Coefficient**	***t*** **value**	***P*** **value**
Non-sarcopenia/Non-obesity	Reference			Reference		
Non-sarcopenia/Obesity	-0.052	-1.40	0.161	-0.087	-2.69	0.007
Sarcopenia/Non-obesity	-0.086	-2.32	0.021	-0.087	-2.65	0.008
Sarcopenia/Obesity	-0.121	-3.33	<0.001	-0.120	-3.74	<0.001
Age				-0.396	-12.33	<0.001
Male sex				0.224	6.92	<0.001
**6MWD**	**Unadjusted model**			Adjusted model*		
	**Coefficient**	***t*** **value**	***P*** **value**	**Coefficient**	***t*** **value**	***P*** **value**
Non-sarcopenia/Non-obesity	Reference			Reference		
Non-sarcopenia/Obesity	-29.87	-2.64	0.008	-40.43	-4.16	<0.001
Sarcopenia/Non-obesity	-34.25	-2.64	0.008	-30.78	-2.82	0.005
Sarcopenia/Obesity	-83.58	-3.41	<0.001	-77.42	-3.61	<0.001
Age				-6.70	-12.97	<0.001
Male sex				58.45	7.17	<0.001

*6MWD* 6-minute walking distance, *SPPB* short physical performance battery

^a^Adjusted for age and sex

### Outcomes

**Fig. 2:**
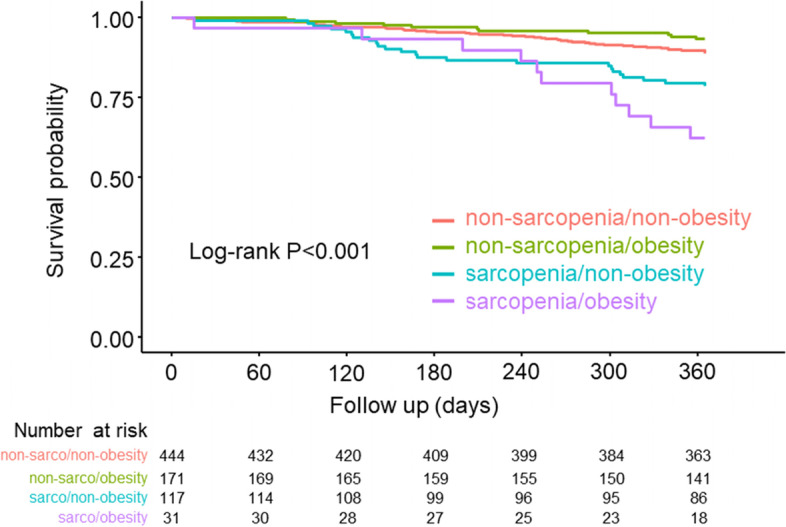
Kaplan–Meier curve for all-cause death based on the presence/absence of sarcopenia and obesity

During the 1-year follow-up period after discharge, 96 (12.3%) all-cause deaths (75 CVD and 21 non-CVD) were observed. Kaplan-Meier curves for all-cause death showed that the sarcopenia/obesity group had significantly lower event-free rates than the other groups (log-rank *P* < 0.001) (Fig. 2). Similarly, sarcopenia/obesity occurrence was related to lower CVD and non-CVD free rates (CVD: log-rank *P* < 0.001; non-CVD: log-rank *P* = 0.001) (Supplemental Fig. 2). Cox proportional hazard analysis revealed that sarcopenia/obesity was an independent prognostic factor of all-cause death even after adjusting for MAGGIC risk score and log-transformed BNP (non-sarcopenia/non-obesity vs. sarcopenia/obesity: hazard ratio, 2.48; 95% confidence interval, 1.22–5.04; *P* = 0.012) (Table 3). When the non-sarcopenia/obesity group was considered as the reference, sarcopenia/obesity occurrence was significantly associated with all-cause mortality after adjustment for MAGGIC risk score and log-transformed BNP (non-sarcopenia/obesity vs. sarcopenia/obesity: hazard ratio, 3.63; 95% confidence interval, 1.52–8.68; *P* = 0.004).

**Table 3:** Cox proportional hazard analysis for all-cause death

	Unadjusted model					Adjusted model*				
	Hazard ratio	95% CI			*P* value	Hazard ratio	95% CI			*P* value
Non-sarcopenia/Non-obesity	1 (reference)					1 (reference)				
Non-sarcopenia/Obesity	0.59	0.30	–	1.13	0.109	0.68	0.35	–	1.33	0.259
Sarcopenia/Non-obesity	2.03	1.24	–	3.31	0.005	1.78	1.06	–	2.98	0.029
Sarcopenia/Obesity	3.74	1.94	–	7.21	<0.001	2.48	1.22	–	5.04	0.012

*CI *confidence interval

Adjusted for Meta-analysis Global Group in Chronic Heart Failure risk score and log-transformed brain natriuretic peptide

## Discussion

This study is the first to investigate the prognostic value of sarcopenic obesity in 779 hospitalized older adults with HF. Our main findings are as follows: 1) the prevalences of sarcopenic obesity and sarcopenia without obesity in hospitalized patients with HF were 3.9 and 15.3%, respectively; 2) when we divided all study patients into four groups based on the presence or absence of sarcopenia and obesity, physical function was more impaired in patients with sarcopenic obesity independent of age and sex; and 3) after adjusting for other prognostic predictors, sarcopenic obesity was found to be an independent predictor of all-cause mortality, unlike the absence of sarcopenia and obesity.

The prevalence of sarcopenia in patients with HF was reported as 19.5% in the SICA-HF study [24], which was comparable to that reported in this study (19.3%). In contrast, the reported prevalence of obesity classified by percentage body fat in patients with HF in Europe is 32.3% [25], which is higher than that reported in our study (26.2%). Moreover, the prevalence of sarcopenic obesity in patients with HF in Europe is 18.5% [7], which is higher than that in our study (4.0%), although direct comparison could not be made because of the use of different criteria for sarcopenia diagnosis. A probable reason for the lower prevalence of sarcopenic obesity in our study was the lower rate of obesity in our patients with HF. Indeed, patients with HF in Western countries are reportedly more obese than those with HF in Asia. In a previous study, when BMI > 30 kg/m^2^ was defined as obesity, 40/808 (5.0%) of patients with HF in Asia were diagnosed as obese [26], whereas 1702/6140 (27.7%) of patients with HF in four other regions (Europe, North America, South America, and Asia) were classified as obese [27]. The definition of low muscle mass also affects the prevalence of sarcopenic obesity. A previous study reported that the prevalence of sarcopenic obesity was higher when the appendicular skeletal muscle mass was adjusted by body weight than by height squared [28]. Another study involving 771 older Taiwanese adults revealed that low muscle mass adjusted by body weight was superior in identifying patients with sarcopenic obesity compared to a height-adjusted method [29]. The use of the appendicular skeletal muscle mass adjusted by body weight would be more appropriate in diagnosing sarcopenic obesity, although the AWGS, which proposed universal criteria for the Asian population, recommends the use of height-adjusted skeletal muscle mass [12].

Several studies have hitherto reported an association between sarcopenic obesity and exercise capacity in older community-dwelling individuals [30]. A previous study involving 2303 community-dwelling residents aged 70–84 years reported lower SPPB scores in residents with sarcopenic obesity than in those with sarcopenia or obesity [30]. Contrariwise, a clinical study including 1468 community-dwelling older Turks showed that the occurrence of sarcopenia alone was significantly associated with impaired activities of daily living compared to the occurrence of sarcopenic obesity [31]. The reason why this result [31] was different from ours was unclear; however, there was a greater proportion of younger (median age, 75 years) and male (68.8%) participants in the previous study [31] than in our study, and different criteria were used for sarcopenia diagnosis (the European Working Group on Sarcopenia in Older People 2). Moreover, the study population—our study included only patients with HF—might have affected the relationship between sarcopenic obesity and exercise capacity. Although a sarcopenia [24, 32] and obesity [33] in patients with HF are individually associated with decreased exercise capacity as evaluated using the SPPB score and 6MWD, there are no reports investigating the impact of sarcopenic obesity on exercise capacity (evaluated using the SPPB score or 6MWD) in patients with HF. Therefore, sarcopenia, obesity, and sarcopenic obesity may all be factors that reduce physical function in patients with HF. Nevertheless, Haykowsky et al. [34] reported that older patients having HF with preserved ejection fraction (HFpEF) had significantly higher total fat and leg fat percentages measured using dual-energy X-ray absorptiometry (DXA), compared to healthy controls, albeit significantly lower total lean mass percentages. In this report, the SPPB scores of older patients with HFpEF were significantly lower than those of the age-matched healthy controls. Furthermore, older patients with HFpEF had significantly reduced total and leg lean mass percentages compared with healthy controls. This is associated with their severely reduced peak oxygen uptake (expressed as absolute or indexed to body mass, total lean mass, or leg lean mass) and physical functional performance compared with healthy controls. Haykowsky et al. also reported that intermuscular fat is a predictor of peak oxygen uptake and 6MWD in older patients with obesity and HF [33]. In addition, they reported that abnormal skeletal muscle composition (abnormal fat infiltration into the thigh skeletal muscle) in patients with HFpEF was associated with a decrease in peak oxygen uptake [35]. The results of these studies suggest that sarcopenic obesity with decreased skeletal muscle mass and increased fat mass contributes to low physical function. Therefore, it may be possible to partially explain the decrease in SPPB score and 6MWD in patients with sarcopenic obesity in this study. It is well-known that higher body FM is associated s better clinical outcomes in patients with HF [36–38]; surprisingly, sarcopenic obesity was associated with worse outcomes in the current study. Although the reason of the discrepancy is uncertain, our study had a few differences from the previous studies. First, our study included older patients (median age, 81 years) than other studies. Moreover, different definitions and cut-off values of FM were used. We defined low FM as FM measured by BIA < 27 and < 38% for men and women, respectively, in the current study. In contrast, Thomas et al. defined low FM as FM measured using DXA ≤8.2 kg/m^2^ in both sexes [36]. Aimo et al. estimated body fat content using age, sex, and BMI, without using instruments [37]. Ohori et al. used DXA to evaluate FM with cut-off values of 25 and 30% for men and women, respectively [38]. These discrepancies might affect the association between FM and prognosis. The diagnosis of sarcopenia is a useful prognostic factor, especially in obese patients because obese patients without sarcopenia had better outcomes in our study. Therefore, we believe that clinicians should evaluate the sarcopenia status in patients with HF and consider whether a careful follow-up or medical intervention may be needed. A previous study on older obese patients with HFpEF reported that caloric restriction diet exposure or aerobic exercise training decreased FM and increased lean mass and peak oxygen consumption [39]. In addition, the muscle mass is directly associated with exercise capacity in patients with HF [40]. Therefore, exposure to resistance and/or aerobic exercise and proper diet with adequate protein intake may improve exercise capacity in patients with sarcopenic obesity having HF. A previous meta-analysis results show an association between sarcopenic obesity occurrence and all-cause mortality, especially in men [41] and older hospitalized patients [6]. However, there was no report on the abovementioned association for hospitalized patients with HF. Akin to previous study findings, we showed that sarcopenic obesity occurrence was associated with all-cause mortality. The results of this study showed a worse prognosis for patients with sarcopenic obesity and sarcopenia without obesity compared to that of individuals with neither sarcopenia nor obesity. These findings suggest that muscle mass could partially explain the obesity paradox [42, 43] in patients with HF, and could be used to stratify the risk both in patients with and without obesity. However, there is no consensus on diagnostic criteria for sarcopenic obesity. The difference in the definition of obesity between Westerners and Asians is a reason why a consensus on a universal cut-off value has not been reached. In the present study, we defined obesity in Asian patients with HF using Baumgartner’s criteria, which have been used in many previous studies on Westerners [44–47], and showed that sarcopenic obesity occurrence was associated with all-cause mortality. Therefore, Baumgartner criteria may be used to define obesity in the diagnosis of sarcopenic obesity in older Asian patients with HF.

There are several limitations in this study. First, in previous studies conducted on HF patients with sarcopenic obesity, muscle mass was measured using DXA [7]. However, in this study, muscle mass was measured using the BIA method. The accuracy of BIA in sarcopenia diagnosis may depend on the accuracy of the equipment used and the measurement conditions, although we previously confirmed excellent inter- and intra-examiner reliabilities in another sub-analysis of the FRAGILE-HF studystudy [48]. Furthermore, different results may be obtained, since the accuracy of BIA instruments was not examined. Second, although the assessment of sarcopenia and obesity was performed before discharge when patients were in a compensated, euvolaemic state, BIA could not be used in this study to calculate the ratio of external cell water to total body water content. Therefore, fluid volume might have affected effectthe appendicular skeletal muscle mass or body FM percentage. Third, some patients were excluded for several reasons, including the presence of an implanted device. Thus, we performed an additional analysis, which included these patients. However, we confirmed that there was no significant prognostic difference between the excluded and included patients. Fourth, our study showed that sarcopenic obesity occurrence was associated with a worse patient prognosis at the time of discharge. Chun et al. reported that resistance exercise exerts benefits on the body composition in obese women with sarcopenia [49]. Our study participants were patients hospitalized for HF, and thus aggressive resistance exercise was not performed. More so, the relationship between post-discharge resistance exercise interventions and changes in body composition and prognosis was not clarified. Fifth, we used the revised Japanese version of the Cardiovascular Health Study criteria for diagnosing low physical activity. However, the criteria were not universal; other methods, such as the use of the simple five-item questionnaire, are reportedly effective in diagnosing low physical activity [50]. Finally, this study was conducted in Japanese older adults with HF; hence, further studies in other populations are required to validate the prognostic value of sarcopenic obesity.

## Conclusion

Sarcopenic obesity was found to be a risk factor for all-cause death and low physical function in older adults with HF. Further studies are needed to establish novel methods for assessing sarcopenic obesity and design interventional studies on treatment strategies.

## Supplementary Information

**Additional file 1: Supplemental Figure 1**. Kaplan–Meier curve for all-cause death based on the excluded and included patients. **Supplemental Figure 2**. Kaplan–Meier curve for cardiovascular death (A) and non-cardiovascular death (B) according to the excluded patients and the study patients.

**Additional file 2: Supplemental Table 1**. Analysis of covariance between sarcopenia/obesity status and physical functions.

## Data Availability

The present study was a post-hoc sub-analysis of the FRAGILE-HF findings. The dataset generated and analysed during the current study is not publicly available due to the sensitivity of personal information; however, it may be available from Yuya Matsue upon reasonable request.

## Abbreviations

6MWD: 6-minute walk distance
AWGS: Asian Working Group for Sarcopenia
BIA: Bioelectrical impedance analysis
CVD: Cardiovascular death
FM: Body fat mass
DXA: Dual-energy X-ray absorptiometry
HF: Heart failure
HFpEF: Heart failure with preserved ejection fraction
IQR: Interquartile range
MAGGIC: Meta-analysis Global Group in Chronic Heart Failure
FRAGILE-HF: Prevalence and prognostic value of physical and social frailty in geriatric patients hospitalized for HF: a multicentre prospective cohort study; SPPB, Short physical performance battery
