# Supplementary material for: Sarcopenic obesity is associated with impaired physical function and mortality in older patients with heart failure: insight from FRAGILE-HF
Source: BMC Geriatr. 2022 Jul 5;22:556. doi: 10.1186/s12877-022-03168-3 (PMC9254413; doi:10.1186/s12877-022-03168-3)
Supplement: Supplementary file 1 — Additional file 1: Supplemental Figure 1. Kaplan–Meier curve for all-cause death based on the excluded and included patients. Supplemental Figure 2. Kaplan–Meier curve for cardiovascular death (A) and non-cardiovascular death (B) according to the excluded patients and the study patients. [file 12877_2022_3168_MOESM1_ESM.docx]

**Supplemental Figure 1. Kaplan–Meier curve for all-cause death based on the excluded and included patients.**


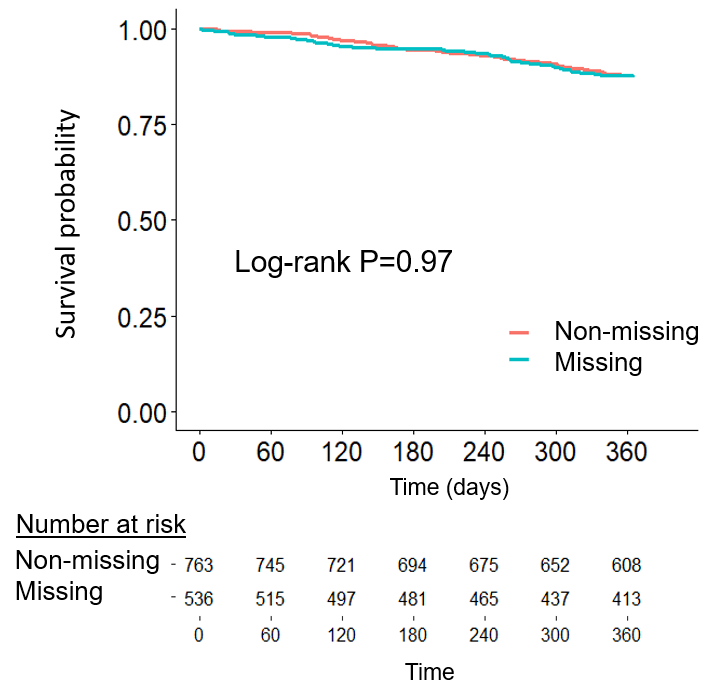


**Supplemental Figure 2. Kaplan–Meier curve for cardiovascular death (A) and non-cardiovascular death (B) according to the excluded patients and the study patients.**


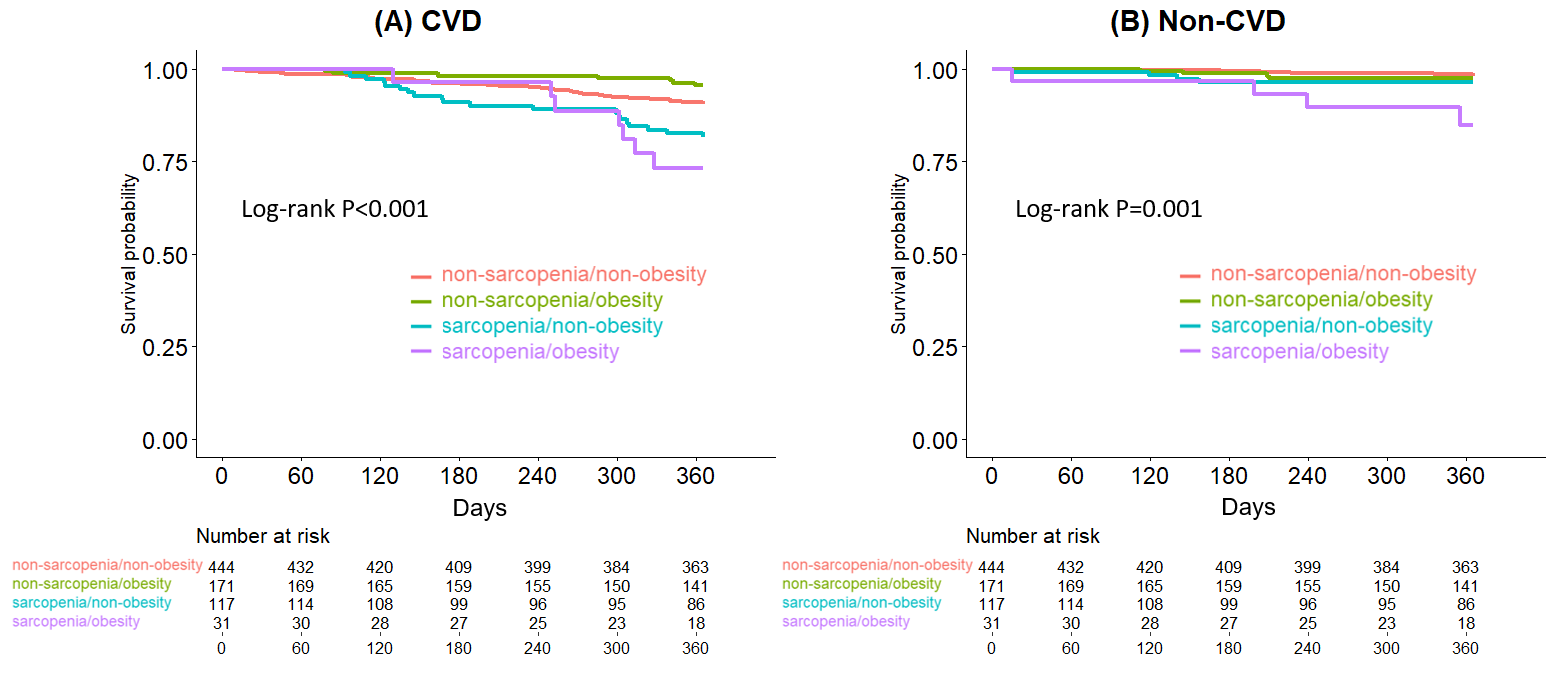


CVD; cardiovascular death
