# Supplementary material for: Sarcopenic obesity is associated with impaired physical function and mortality in older patients with heart failure: insight from FRAGILE-HF
Source: BMC Geriatr. 2022 Jul 5;22:556. doi: 10.1186/s12877-022-03168-3 (PMC9254413; doi:10.1186/s12877-022-03168-3)
Supplement: Supplementary file 2 — Additional file 2: Supplemental Table 1. Analysis of covariance between sarcopenia/obesity status and physical functions. [file 12877_2022_3168_MOESM2_ESM.docx]

**Supplemental Table 1. Analysis of covariance between sarcopenia/obesity status and physical functions**

| SPPB |  | Adjusted for age and sex | | | | |  |  |
| --- | --- | --- | --- | --- | --- | --- | --- | --- |
|  |  | LS means | Standard error | Group difference (95% CI) | *P* value | | |  |
|  |  |  |  |  |  |  |  |  |
| Non-sarcopenia/Non-obesity |  | 8.69 | 0.12 | Reference | Reference | | |  |
| Non-sarcopenia/Obesity |  | 7.98 | 0.20 | 0.70 (0.12–1.31) |  | 0.012 | |  |
| Sarcopenia/Non-obesity |  | 7.87 | 0.24 | 0.82 (0.13–1.51) |  | 0.013 | |  |
| Sarcopenia/Obesity |  | 7.00 | 0.47 | 1.68 (0.42–2.95) |  | 0.004 | |  |
|  |  |  |  |  |  |  | |  |
|  | | | | | | |  |  |
|  |  | Adjusted for age and sex | | | | |  |  |
| 6MWD |  | LS means | Standard error | Group difference (95% CI) | *P* value | | |  |
|  |  |  |  |  |  |  |  |  |
| Non-sarcopenia/Non-obesity |  | 281.3 | 5.1 | Reference | Reference | | |  |
| Non-sarcopenia/Obesity |  | 240.8 | 8.3 | 40.4 (15.4–65.4) | < | 0.001 | |  |
| Sarcopenia/Non-obesity |  | 249.5 | 10.0 | 31.8 (2.8–60.8) |  | 0.025 | |  |
| Sarcopenia/Obesity |  | 203.9 | 20.7 | 77.4 (22.2–132.6) |  | 0.002 | |  |
|  |  |  |  |  |  |  | |  |
| 6MWD, 6-minute walk distance; CI, confidence interval; LS, least square; SPPB, short physical performance battery | | | | | | |  |  |
|  |  |  |  |  |  |  |  | |
|  |  |  |  |  |  |  |  | |
